# Propranolol enhanced the anti-tumor effect of sunitinib by inhibiting proliferation and inducing G0/G1/S phase arrest in malignant melanoma

**DOI:** 10.18632/oncotarget.22696

**Published:** 2017-11-25

**Authors:** Xinwei Kuang, Min Qi, Cong Peng, Chengfang Zhou, Juan Su, Weiqi Zeng, Hong Liu, Jianglin Zhang, Mingliang Chen, Minxue Shen, Xiaoyun Xie, Fangfang Li, Shuang Zhao, Qingling Li, Zhongling Luo, Junchen Chen, Juan Tao, Yijing He, Xiang Chen

**Affiliations:** ^1^ Department of Dermatology, XiangYa Hospital, Central South University, Changsha, China; ^2^ Hunan Key Laboratory of Skin Cancer and Psoriasis, Changsha, China; ^3^ Department of Plastic and Cosmetic Surgery, XiangYa Hospital, Central South University, Changsha, China; ^4^ Department of Clinical Pharmacology, XiangYa Hospital, Central South University, Changsha, China; ^5^ Department of Rheumatology, XiangYa Hospital, Central South University, Changsha, China; ^6^ Department of Pathology, XiangYa Hospital, Central South University, Changsha, China; ^7^ Department of Dermatology, Affiliated Union Hospital, Tongji Medical College, Huazhong University of Science and Technology, Wuhan, China

**Keywords:** malignant melanoma, sunitinib, propranolol, combination treatment, cell cycle

## Abstract

Both sunitinib, a multi-target tyrosine kinase inhibitor (TKI) and propranolol, a non-selective β-blocker, have proven therapeutic effects on malignant melanoma (MM). This study reports a synergistic effect of propranolol and sunitinib upon A375, P8 MM cell lines and mice xenografts. Cell viability assays detected a significant decrease of sunitinib IC50 in combination with propranolol, which was confirmed by a colony formation assay. Western blot showed that propranolol and sunitinib combination significantly down-regulated phospho-Rb, phospho-ERK, Cyclin D1, and Cyclin E, but had no effect on Bax, Bcl-2, or cleaved PARP expression. The average tumor size of propranolol and low-dose sunitinib (Sun L) combination treated mice was reduced and similar to high-dose sunitinib treated A375 xenografts. The Ki67 index was significantly reduced in propranolol and Sun L combination treated group compared with single Sun L treated group. This synergistic effect between propranolol and sunitinib to inhibit MM proliferation was through suppressing ERK/Cyclin D1/Rb/Cyclin E pathways and inducing G0/G1/S phase arrest, rather than by inducing tumor cell apoptosis.

## INTRODUCTION

Sunitinib a multi-target tyrosine kinase inhibitor (TKI) is currently approved by U.S. F.D.A. for treating advanced renal cell carcinoma, imatinib-resistant, or intolerant gastrointestinal stroma tumor (GIST), and advanced pancreatic neuroendocrine tumors [1]. Recent studies have reported that sunitinib controls c-KIT or non c-KIT mutated malignant melanoma (MM) [2–6]. However, sunitinib is not clinically widely used on solid tumors due to the low efficacy [6–9]. How to improve the efficacy of sunitinib in solid tumor remains a big challenge for oncologists’ world widely. Propranolol, a non-selective β-blocker, is widely used for hypertension. It has been found to be safe and effective in treating large infantile hemangioma [10, 11]. Follow-up studies suggest that it can also be used to treat the malignant tumors such as non-small-cell lung cancer, breast cancer, colorectal cancer, gastric cancer and neuroblastoma cancer [12–20]. Recently, propranolol has been found to inhibit MM proliferation *in vitro* and *in vivo* [21, 22] and prolong MM patient survival [23]. Propranolol shows synergistic effects with various chemotherapies and tumor targeting medications to obtain an anti-tumor effect, including a propranolol-TKI combination treatment [24–30]. However, the underlying mechanism of how propranolol enhanced the efficacy of TKIs remained unclear. This study ascertained that if propranolol would increase sunitinib efficacy in MM and aimed to explore potential mechanism.

## RESULTS

### Combined propranolol-sunitinib treatment (C-PST) inhibited cutaneous-acral MM proliferation

**Figure 1:**
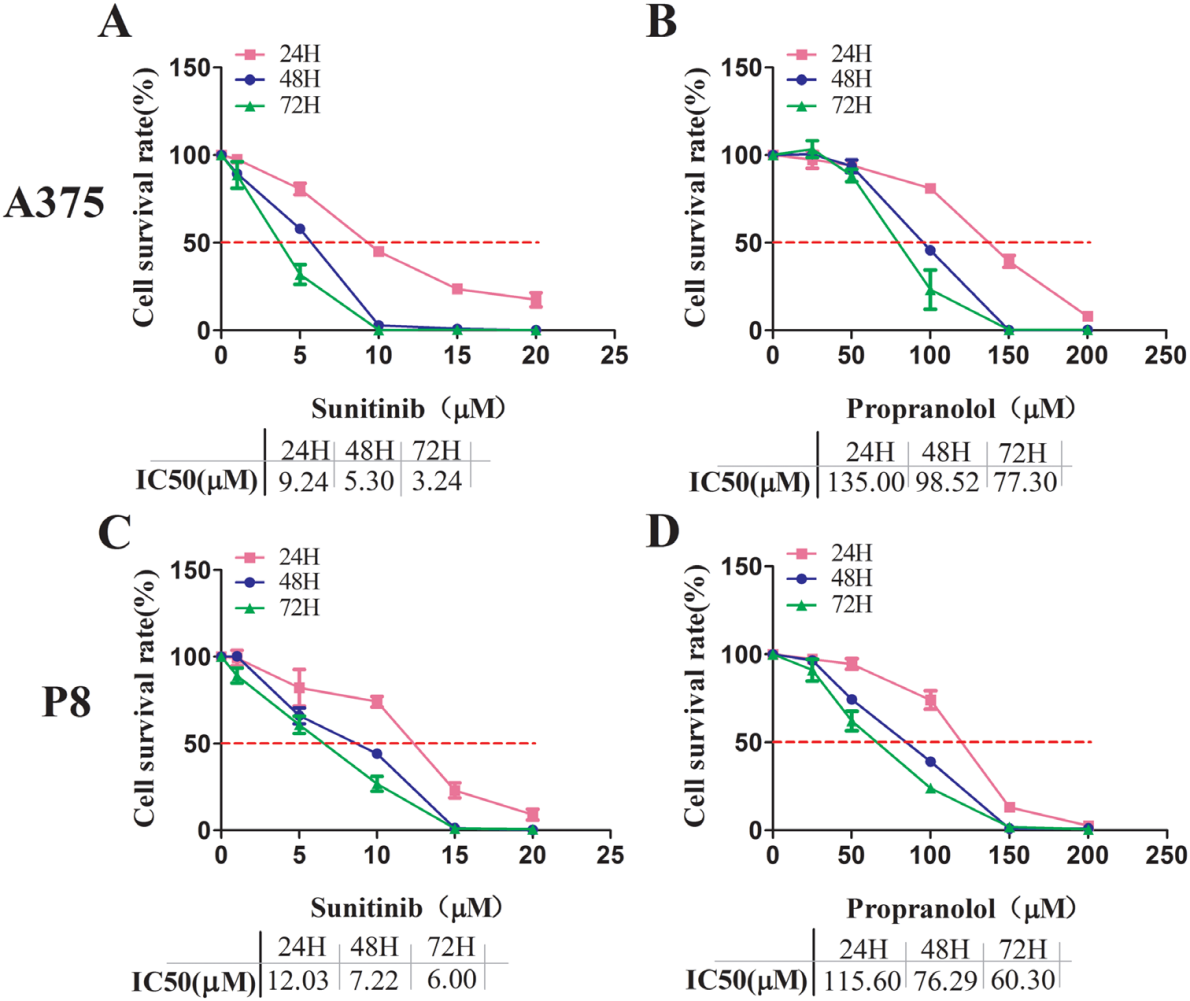
Sunitinib and propranolol effects on cell proliferation in A375 and P8 melanoma cell lines **(A-D)** MTS assay measured cell viability after increasing suninitib (1, 5, 10, 15, 20 µM) and propranolol (25, 50, 100, 150, 200 µM) concentrations at 24, 48, and 72H MTS. Relative growth rate was calculated as the ratio of treated to untreated cells at each dose for each replicate. Sunitinib IC50 and propranolol, after incubation for 24, 48, and 72H, was 9.24, 5.3, 3.24µM, and 135, 98.52, 77.3 µM, respectively, in the A375 cell lines. It was 12.03, 7.22, 6.00 µM and 115.6, 76.29 60.3 µM in P8 cell lines respectively.

**Figure 2:**
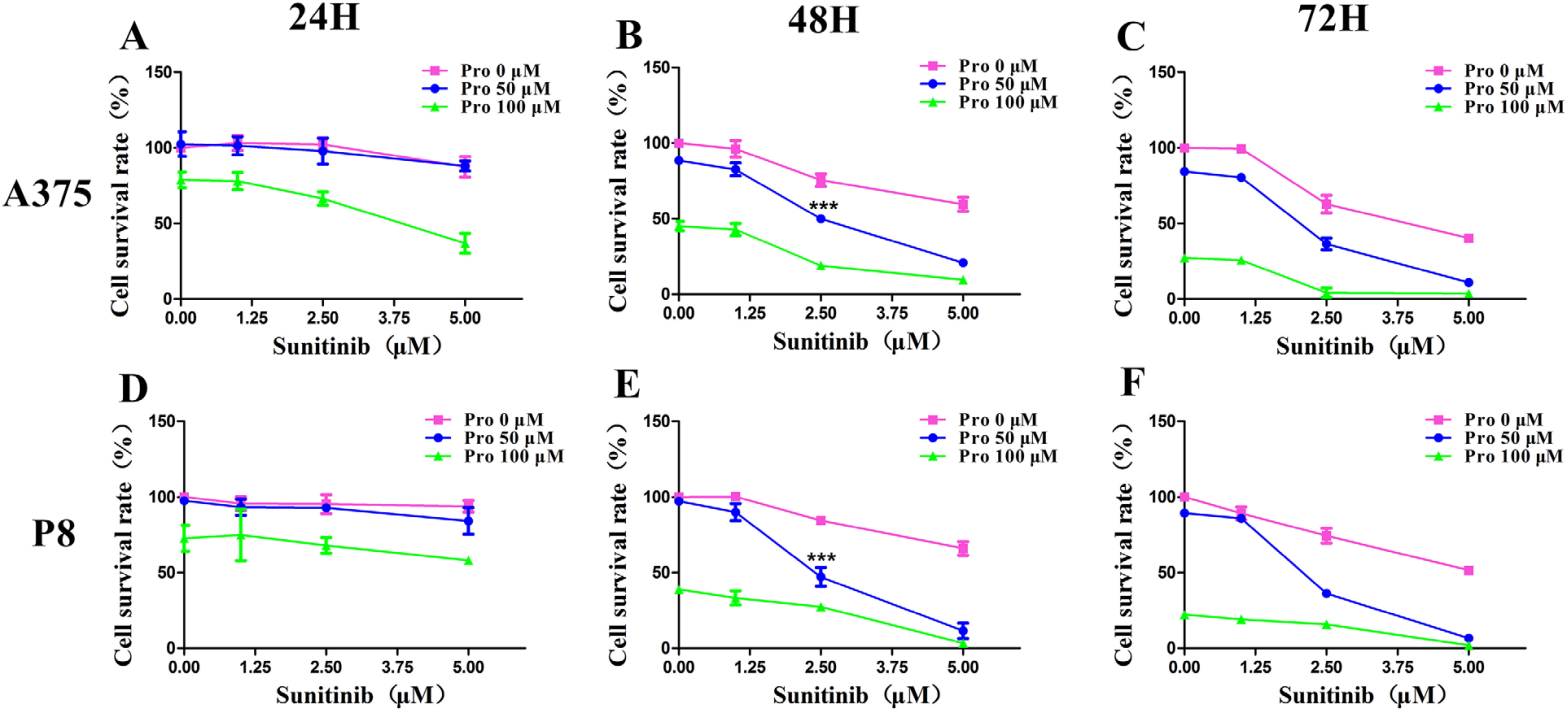
C-PST effects on cell proliferation in melanoma cell lines **(A-F)** An MTS assay calculated survival rate from the combined treatment for sunitinib (1, 2.5, 5 µM) and propranolol (50, 100 µM) concentrations at 24, 48, 72H. Results are presented as mean ± SD. Significant differences were evaluated using one-way ANOVA, and the asterisk (^***^) indicates a significant difference compared the same concentration of sunitinib or propranolol alone with sunitinib 2.5 µM and propranolol 50 µM combination group using Dunnett’s multiple comparison test(P<0.001). Pro, propranolol.

A cell viability assay demonstrated that sunitinib 1-20 μM and propranolol 25-200 μM significantly reduced the survival rate of two MM cell lines in a concentration and time dependent manner. The data at 24, 48, and 72 hours were shown in respectively (Figure 1. The greatest inhibition on cell proliferation in incubated MM cell lines was detected by using a combination of propranolol 50 μM and sunitinib 2.5 μM. The survival rate of A375 cell line and P8 cell line was 49.9±1.1% and 47.2±2.8% at 48 hours respectively (Figure 2B and 2E). This data demonstrated that the IC50 of sunitinib in propranolol-sunitinib combination was decreased by 53% and 65% respectively on A375 and P8 cell lines compared with single sunitinib treatment.

**Figure 3:**
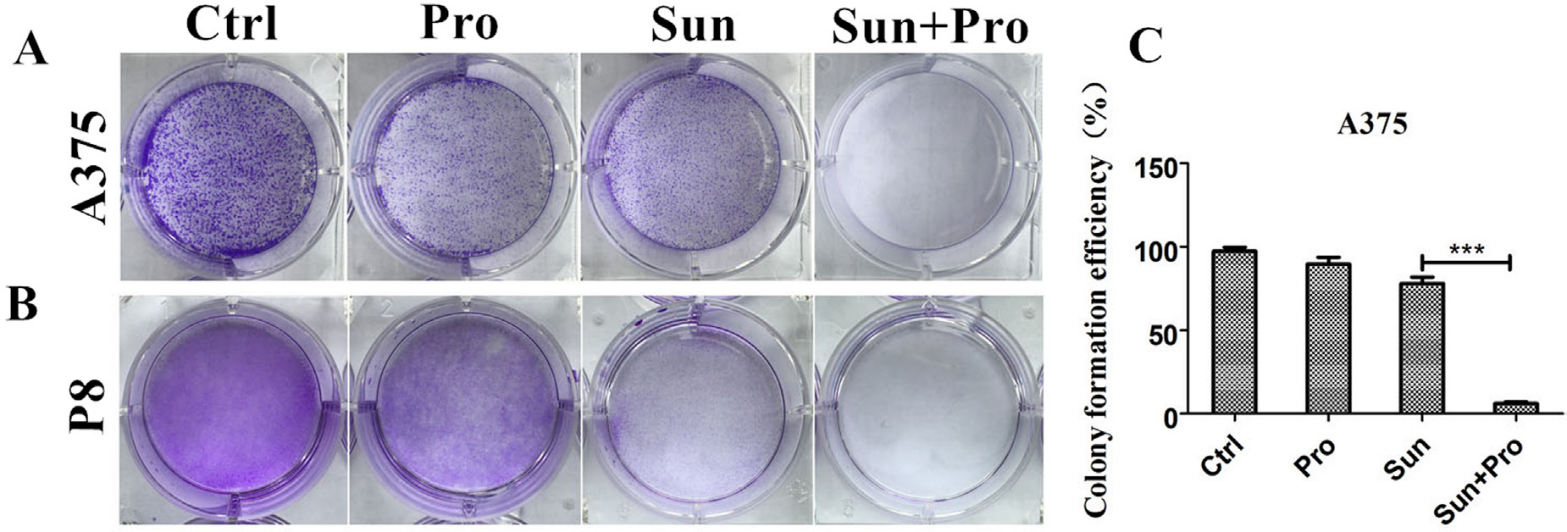
C-PST significantly reduced colony growth in A375 and P8 cell lines A colony formation assay measured A375 cell **(A)** and P8 lines **(B)**. Cell seed concentration was 2^*^10^3^ and 1^*^10^3^ cells. Cultivation lasted 7 and 9 days respectively. PBS and DMSO, in equal volumes, were mixed as negative controls and treated with propranolol, sunitinib, and the C-PST. **(C)** Colony formation efficiency is described in A. Error bars represent the standard deviation. Results are presented as mean ± SD, ^***^P<0.001.

The formation of cell colony in the A375 cell line was reduced from 78±3.8% to 6±1.1% by C-PST compared with single sunitinib treatment measured by colony formation assay (Figure 3A and 3C). In P8 cell line, no cell colony was formed in C-PST treated group (Figure 3B, and Supplementary Figure 1). In the following experiment, the dosage of C-PST was fixed at propranolol 50 μM in combination with sunitinib 2.5 μM.

### C-PST induced G0/G1/S phase arrest in MM cell lines

**Figure 4:**
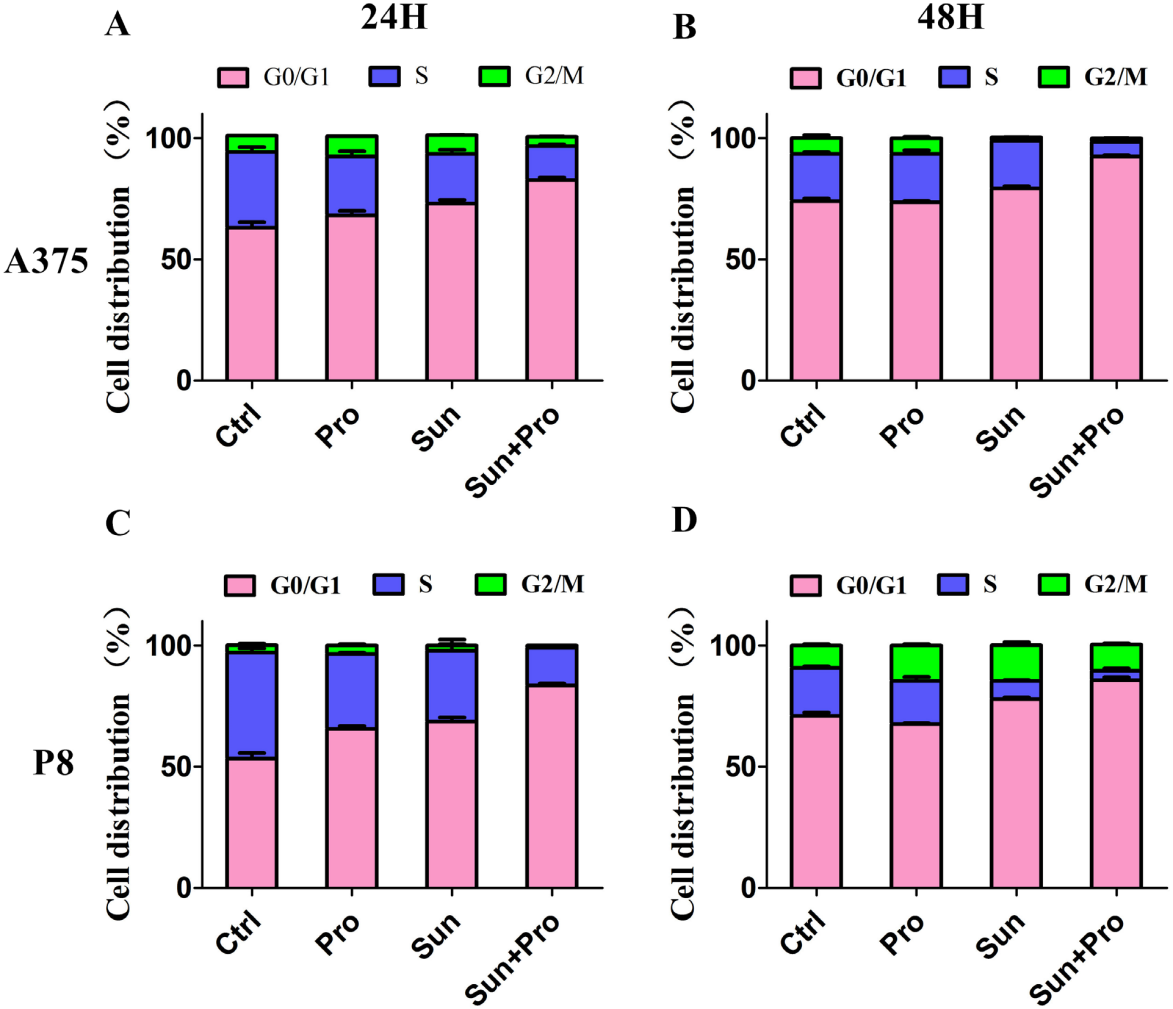
Cell-cycle analysis (G0/G1, S, and G2/M) of A375 and P8 cell lines **(A-D)** Flow cytometric analysis of A375 and P8 human MM cells exposed to propranolol 50 µM, sunitinib 2.5 µM, and C-PST after 24 and 48H cultivation to establish cell percentages in G0/G1, S, and G2/M phases of the cell cycle. Data are presented as mean ± SD.

**Figure 5:**
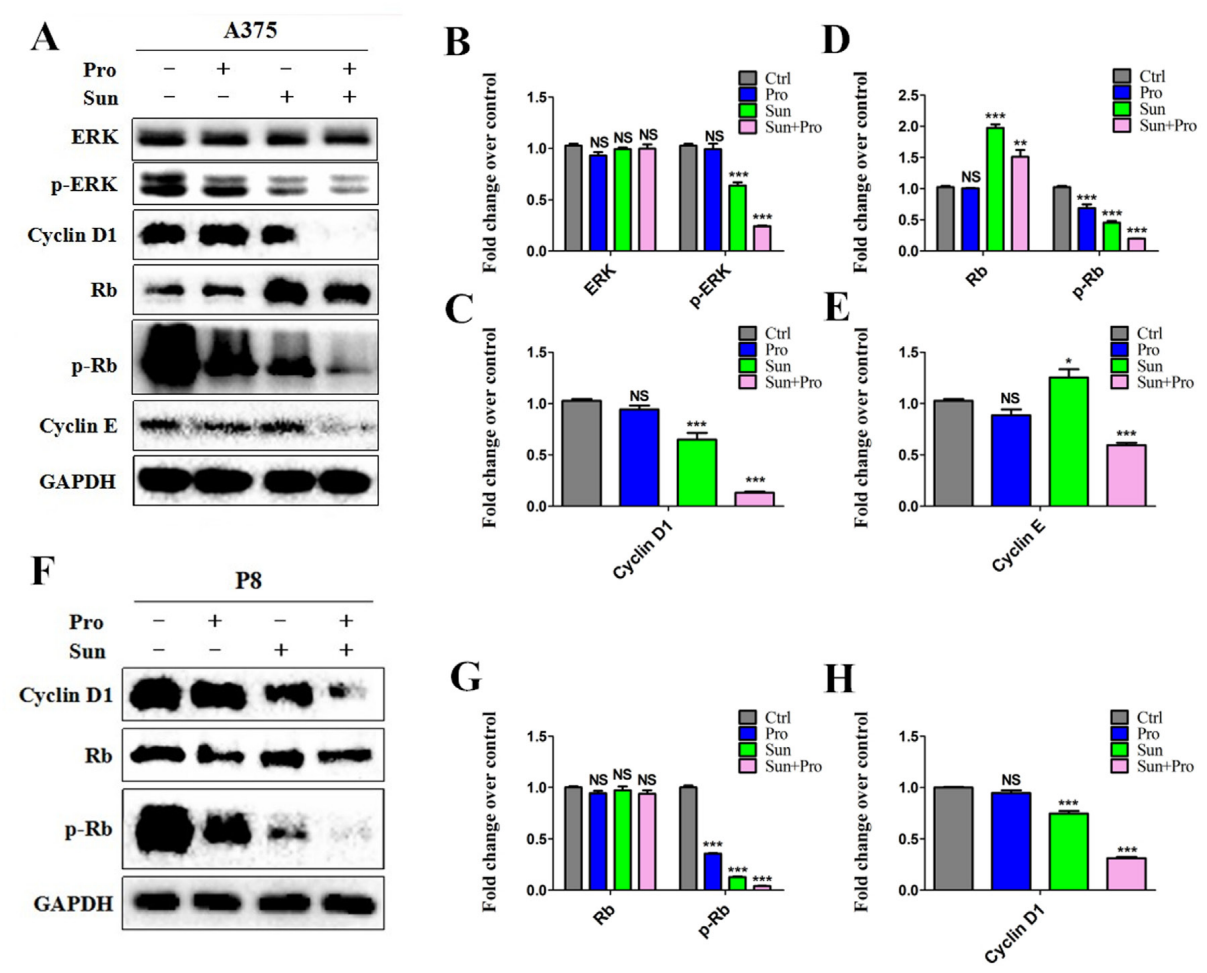
Cell cycle related western blot analysis of C-PST in MM cells **(A)(F)** C-PST suppressed ERK1/2 and Rb phosphorlate levels. Cyclin D1 and Cyclin E expression levels were suppressed in both A375 and P8 cell lines. ERK, p-ERK, Cyclin D1, Rb, p-Rb, and Cyclin E protein fold levels changed in propranolol 50 µM, sunitinib 2.5 µM, the C-PST and the control groups in **(B-E)** A375 cells and **(G, H)** P8 cells. Results were determined by three independent experiments and normalized by total protein level. Data are presented as mean ± SD. ^*^P<0.05, ^**^P<0.01 and ^***^P<0.001 vs. Ctrl.

Cell cycle progression was delayed by C-PST compared with the single group. After C-PST the cell amount was significantly increased in G0/G1 phase and decreased in S phase at 24, 48 hours in A375 (Figure 4A and 4B) and P8 (Figure 4C and 4D). No significantly changes were seen in the G2/M phase (P>0.05) (Supplementary Table 1). These findings suggested that the C-PST inhibited MM cell lines by arresting cell progression at the G0/G1 and S phases. The phospho-ERK1/2, phospho-Rb, Cyclin D1, and Cyclin E expression were significantly down-regulated in the A375 (Figure 5A) and P8 cell lines (Figure 5F) after C-PST treatment for 24 hours. Fold changes of the expression were calculated for ERK, p-ERK, Cyclin D1, Rb, p-Rb, and Cyclin E proteins treated by propranolol, sunitinib, C-PST and control group in (Figure 5B-5E) A375 cells and (Figure 5G, 5H) P8 cells. The expression of p-ERK, Cyclin D1, p-Rb, and Cyclin E proteins were statistically different between single sunitinib and C-PST group (P<0.001), meanwhile; the expression of ERK and Rb showed no significant statistical differences(P>0.05).

### C-PST inhibited *in vivo* MM growth

**Figure 6:**
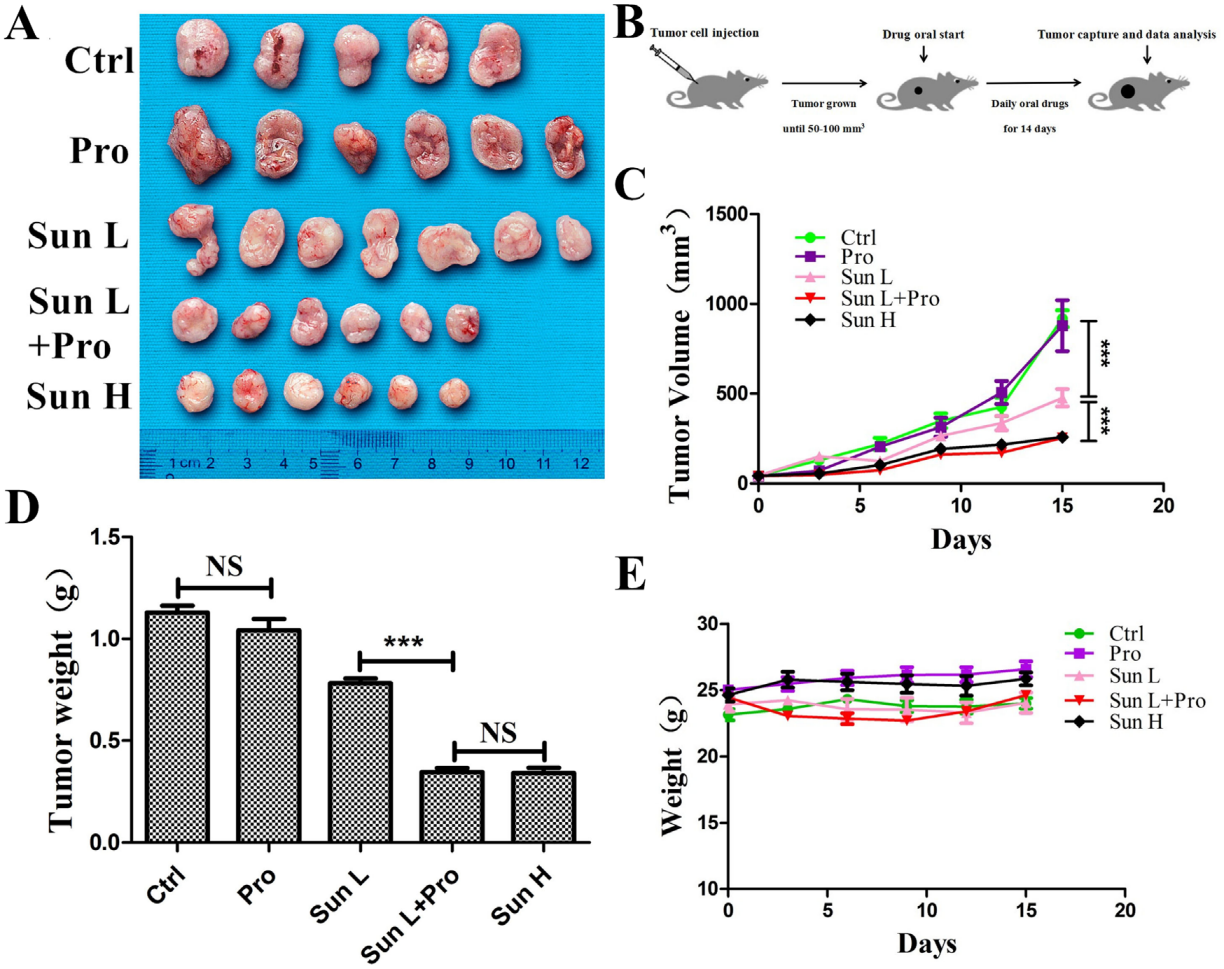
C-PST inhibited *in vivo* tumor development **(A)** A375 untreated mice (PBS and citrate buffer mixed group) xenograft excised tumors and mice treated with propranolol 2mg/k^*^day: Pro, low-dose sunitinib 40mg/kg^*^day: Sun L, Sun L+Pro, high-dose sunitinib 80mg/kg^*^day: Sun H. **(B)** Schematic plan for the administration of control (PBS and citrate buffer mixed group), Pro, Sun L, Sun L+Pro or Sun H to tumor-bearing mice. **(C)** Tumor growth curves measured by average volume of 5 to 7 tumors for each group. **(D)** All the tumors were removed and measured by average weight for each group at day 15. **(E)** A375 model mice body weights were measured at the beginning of the test and every three days there after. Results are presented as mean ± SD, ^***^P<0.001.

**Figure 7:**
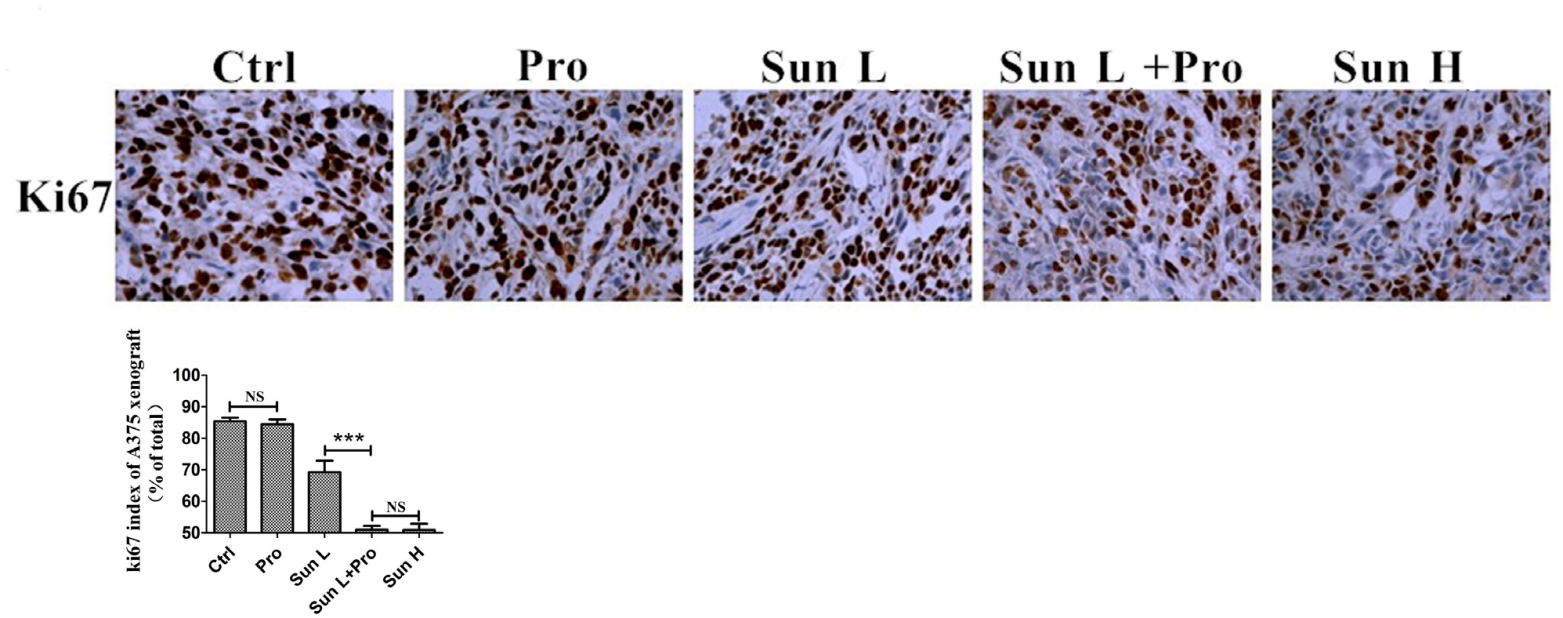
C-PLST inhibited xenograft tumor development **(A)** An immunohistochemistry assay assessed Ki67. **(B)** The Ki67 index. Results are presented as mean ± SD, ^***^P<0.001.

A375 MM cell was engrafted into BALB/C nude mice. About 70-80% of mice developed solid tumors in 5-7 days. Mice were divided into five groups including: blank control (citrate buffer and PBS); low-dose sunitinib (40mg/ kg^*^day, Sun L); Sun L and propranolol (2mg/ kg^*^day) combination (C-PLST); high-dose sunitinib (80mg/ kg^*^day, Sun H); and propranolol (2mg/ kg^*^day). All mice were treated for two weeks. The average tumor size in C-PLST group was smaller than Sun L group (253.78.41±24.57mm^3^ vs. 477.41±48.11mm^3^, P<0.0001, Figure 6A, 6C). The tumor size was similar in C-PLST group and Sun H group at 14 days in A375 xenografts (253.78±24.57mm^3^ *vs.* 259.34±27.82 mm^3^, P>0.05). The wight of tumors were also significantly different between Sun L and C-PLST groups (0.346±0.019 g vs. 0.781±0.024 g, P<0.0001, Figure 6D). Meanwhile, the mice in all treatment groups were not lost body weight and got a slight increase (Figure 6E), which suggested the low toxicity of this combination of medicines. Meantime, in A375 xenografts (Figure 7), the Ki67 expression in C-PLST group was significantly lower than Sun L group (50.55±1.25% vs. 67.88±4.53 %, P<0.01), however, no difference from Sun H group (50.55±1.25% vs. 51.69±1.68 %, P>0.05). This result suggested C-PLST could significantly inhibit the proliferation of MM *in vivo*.

## DISCUSSION

Propranolol was commonly used for hypertension and arrhythmias; moreover, the anti-tumor effect has been widely reported recently [10–22]. Interestingly, propranolol also displayed synergistic effect on other anti-tumor drugs [24–30]. For instance, Wei, et al.[28] investigated the combined administration of propranolol with vemurafenib (a BRAF V600E inhibitor) in thyroid cancer. The two drugs effectively controlled the tumor growth by blocking the β_2_-AR, and then consequently suppressing the expression of p-Akt, p-mTOR, Bcl-2, cyclin D1, HK2 and GLUT1, which indicated that propranolol could enhance the efficacy of vemurafenib, a member of TKIs. However, whether propranolol could also enhance the efficacy of other TKIs, such as sunitinib (VEGF inhibitor) is still unclear.

In the present study, we found that propranolol enhanced sunitinib efficacy by inducing cell cycle arrest at G0/G1/S phase and inhibiting the MM proliferation in melanoma, rather than inducing tumor cell apoptosis (Supplementary Figure 2). Moreover, in A375 xenografts low-dose propranolol in combination with low-dose sunitinib achieved similar tumor control effect to high-dose sunitinib without obvious side effects. Immunohistochemistry of the tumor sections confirmed the enhanced anti-proliferation effect of propranolol on sunitinib measured by Ki67. Meantime, the expression of apoptosis-related proteins, including cleaved-PARP, bax, and bcl2 was not significantly different among propranolol, sunitinib or C-PST groups measured by WB assay (Supplementary Figure 2). Cell morphological changes were also observed (Supplementary Figure 3) which suggested no apoptosis had occurred by using C-PST. This finding highlighted a potential combination therapy of propranolol and sunitinib in the clinic.

In cancer adjuvant therapy, an increasing number of retrospective clinical studies demonstrated the potential benefit from β-blockers in some patients [19, 23, 31–35], such as prostate cancer, non-small-cell lung cancer, breast cancer, hepatocellular carcinoma, ovarian cancer and melanoma. However, inconsistent effectiveness was always found in using β-blockers, mainly due to the following four reasons [36]: 1. the duration time of using β-blockers, 2. the types of β-blocker and subtype of β-AR, 3. the different clinical end point, 4. different tumor heterogeneity and race diversity. Though it has many controversial reports, these findings suggest a role of propranolol in cancer treatment, especially the breast, melanoma and prostate cancer.

β-ARs belong to G protein-coupled receptors family which respond to Norepinephrine (NE) and epinephrine (E), which were widely studied to explore their role of tumor promoting. Liu et al. [37] verified that E promotes esophageal squamous cell carcinoma proliferation by β-AR induced MEK/ERK/COX-2 pathway. In addition, β1 or β2 selective antagonists could abolish HKESC-1 cell proliferation and down regulate phosphorylated ERK1/2 level along with the expression of COX-2. Liao, et al. [17] identified that propranolol promoted cell apoptosis and induced cell cycle arrest via the inhibition of β-ARs and NF- kB in gastric cancer, but the specific mechanism was not clear. Later, Zhang et al. [38] found Ras/Akt/NF-kB pathway was responsible for G1/S phase arrest induced by β_2_-AR blocker (ICI-118,551) in pancreatic cancer. Cyclin D1 and cyclin E were key proteins responsible for cell cycle, ICI-118,551 could inhibit the expression of cell cycle proteins, and it also has a better effect on cell proliferation [38]. These data suggested that, in different cancer cell lines, signal pathways of tumor behaviors could be different, which remind us of exploring other possible mechanisms.

Although there were many options for MM treatment, targeted therapy displayed promising efficacy in clinic, especially BRAF V600E inhibitors. However, other members of TKIs failed to achieve a satisfactory effect in MM such as sunitinib, a VEGF inhibitor. Combination therapy containing propranolol with TKIs may improve the efficacy of TKIs mentioned above [28]; however, the underline mechanism is still unclear. Until now, two reports demonstrated that propranolol could completely abolished the chronic stress-promoted tumor growth in mice MM model and colorectal cancer model through a β-AR dependent pathway and might enhance the sunitinib anti-tumor effects via β-adrenoceptor-cAMP-PKA signaling pathway [29, 30]. These findings provided strong evidence and possible mechanisms for our speculation in combination propranolol with TKIs, and suggested a tendency to extend to more drugs.

What’s more, a few reports showed other synergistic effects of propranolol in combination with other drugs, such as vinblastine, methotrexate, paclitaxel, etoposide, cyclophosphamide, and paclitaxel [24–27]. In triple-negative breast cancer models, Rico M, et al. [39] found that a combination of propranolol and metformin provides a new anti-tumor treatment by decreasing proliferation, mitochondrial activity, migration, and invasion. After examining the immunoregulatory function of propranolol, Ashrafi S et al [40] found that propranolol could play as an adjuvant to tumor vaccine to suppress tumor growth by modulating cytokine pattern in tumor microenvironment.

In conclusion, this study has provided a new treatment option of propranolol in combination with sunitinib for malignant melanoma control in addition to its primary anti-tumor function. Combined administration of propranolol with other TKIs or more anti-tumor drugs may also achieve enhanced anti-tumor effect, which worth further investigation.

## MATERIALS AND METHODS

### Cell lines and reagents

The A375 cell line derived from chronic sun induced damage (CSD) cutaneous MM, was obtained from American type culture collection (ATCC). A375 cell line was cultured in DMEM medium (BI, China) supplemented with 10% FBS(BI, China) at 37°C and 5% CO_2_ in tissue culture incubator.

### Human MM primary cultures

Patient 8 cell line (P8) was derived from surgical resection samples of acral MM from a patient. Written informed consent was obtained from the patient. P8 cell line was cultured in DMEM medium (BI, China) supplemented with 10% FBS (Gibco, Life Technologies Australia) at 37°C and 5% CO_2_ in tissue culture incubator. MM specific antigens were detected via western blotting for CD31, CD63, CD166 and CD146 confirmed that the cells were melanocytes. Tumor tissues were mechanically dissociated with a small tissue chopper prior to sequential enzymatic digestion in 2 mg/ml Collagenase (Sigma-Aldrich, Schnelldorf, Germany) and 1 mg/ml Dispase I (Gibco/Invitrogen, Carlsbad, CA) in DMEM for 30min at 37°C. Cells were filtered (100 μm cell strainer) to obtain a single cell suspension and re-supplemented by phosphate-buffered saline, thereafter centrifuged at 1000 r.p.m. for 5 minutes. Pellet was re-suspended in DMEM with 10% FBS and then cultured in 75 cm^3^ plastic flasks (Themo Fisher Scientific, China).

### Cell viability assays

The half-maximal inhibitory concentration (IC50) value and survival rate(%) were determined by MTS assay (Promega (Beijing) Biotech Co., Ltd. G111B). A375 and P8 were respectively plated in 96-well plates at a density of 3×10^3^ and 5^*^10^3^, treated with 1, 5, 10, 15, 20 μM sunitinib (sunitinib malate, S1042, Selleck, USA) and were treated with propranolol (propranolol hydrochloride, P0884, Sigma-Aldrich, U.S.A) for 24, 48 and 72 hours. Cells were incubated with 10% of MTS: PMS (20:1) mixed reagents 2-4 hours. Fluorescence of each plate was measured using a spectrophotometer at emission 490 nm (Spectra MAX Gemini EM, Molecular Devices).

### Colony formation assays

For colony formation assays, A375 and P8 cells were seeded in a 6-well plate at a density of 2^*^10^3^ and 1^*^10^3^ cells per well and cultured for 7 and 9 days respectively. The dishes were photographed, and the total colony numbers and types of colonies on the plates were evaluated under a microscope. Colonies with > 50 cells were scored. Colony formation efficiency was determined by counting the combination treated group colonies stained with crystal violet solution.

### Cell cycle analysis by flow cytometry

A375, P8 cell lines were cultured at a density of 50 percent in 60 mm^2^ culture dishes (Corning, China) and were treated with sunitinib, propranolol and combination therapy of sunitinb and propranolol after 24 and 48 hours. Cells were then harvested, washed, fixed overnight in 70 % ethanol at 4°C, after washed with PBS, digested with RNase A, stained with propidium iodide (PI). Cells were then processed for flow cytometry analysis by using BD FACSCanto II (Becton, Dickinson and Company, USA). Then, analysis and mapping by flowjo 10.

### Western blot analysis

Western blot analysis was performed on cell extracts of A375 and P8 cell lines treated with sunitinib 2.5 μM, propranolol 50 μM and combination therapy of sunitinib and propranolol for 24, 48 and 72 hours. Cells were lysed in RIPA buffer (Beyotime, China) supplemented with phenylmethylsulphonyl fluoride (Sigma), fresh protease and phosphatase inhibitors (Sigma), and protein concentrations were determined by a BCA Protein Assay Kit (Thermo). Western blotting was performed as described previously. The protein boiled for 10 minimums in loading buffer before resolving by SDS-PAGE. After saturation in Tris-buffered saline supplemented with 5% milk or BSA, the membranes were incubated with antibodies overnight at 4°C. The blots were detected by an imaging system (Bio-Rad, USA). Antibodies specific for the following proteins were ERK1/2 (rabbit, 4695), phospho-p42/44-ERK (rabbit, 4370), Rb (mouse, 9309), phospho-ser807/811-Rb (rabbit, 8516), Cyclin D1 (rabbit, 2922), Parp (rabbit, 9532), purchased from Cell Signaling Technology, and Cyclin E (mouse, 4132, Santa Cruz). The antibodies specific for Bax (rabbit, 50599-2-Ig) and Bcl-2 (rabbit, 12789-1-AP), were purchased from Proteintech. The antibody specific for GAPDH (mouse, clone 6C5, MAB374) was purchased from Millipore. Quantification of the bands was done with Image J.

### Xenografts of human MM

The five-six-week old NOD/SCID male mice (Hunan Silaike experimental Animals Inc, China) were injected subcutaneously with 1^*^10^6^ living MM cells that was maintained in DMEM with 0.5% FBS and 15% Matrigel (BD Bioscience, Franklin Lakes, NJ) in the right flank. A solid tumor develops in A375 xenografts within one- two weeks.

### *In vivo* combination therapy

The NOD/SCID mice with xenografts were randomly divided into five groups after the tumor volume reached 50-100 mm^3^. According to the previous studies [20–22], we choose the dose of propranolol 2mg/ kg^*^day in these studies, which is the lowest dose mentioned before and a good negative control *in vivo*. Meanwhile, mice received a daily oral of sunitinib at the dose of 40mg/ kg for two weeks, and combination therapy of sunitinib 40mg/ kg^*^day and propranolol 2mg/ kg^*^day. We set sunitinib 80mg/ kg^*^day group as the positive control, and the blank control was treated with the same volume of citrate buffer (0.1 mol/L, pH=3.5) and PBS. Tumor volume was measured by length (l), width (w), and height (h) twice a week with avernier caliper and tumor size was calculated by the modified ellipsoidal formula (Tumor volume =π/6× l × w × h). The mice were sacrificed at the end of two-week treatment. The xenografts were removed, and then snap-frozen in liquid nitrogen. Paraffin embedded tumor blocks were prepared for further analysis at the same time.

### Immunohistochemical analysis

The slide was stained according to the manufacturer’s protocol. Briefly, the slide was baked at 60°C for 2 hours, dewaxed in turpentine and rehydrated in a graded ethanol series, and then treated with 3% hydrogen peroxide for 10 minimums to inhibit endogenous peroxidase. The slide was pretreated in a pressure cooker with Antigen Retrieval Solution(in 0.01 M sodium citrate buffer, pH 6.0) for 3 minimums. Tissue sections were then incubated with Peroxidase Blocking Solution (S2023, Dako) for 15 minimums and Protein Block (X0909, Dako) for 20 minimums. Primary antibody specific for Ki67 (anti-rabbit 1:400, ab16667, Abcam) were applied, and the slides were incubated overnight at 4°C. Signals were visualized rabbit HRP-conjugated secondary antibody (K4003, Dako) and a haematoxylin (MHS32, Sigma) counterstain.

### Statistical procedures

Data were expressed as mean ± SD. One-way ANOVA and Dunnett's multiple comparison test were used to determine the statistical differences. A P value of less than 0.05 was considered statistically significant, with the analysis and mapping by Graphpad Prism software (GraphPad Software, Inc., version 5.0).

## SUPPLEMENTARY MATERIALS FIGURES AND TABLE


