# Supplementary material for: Propranolol enhanced the anti-tumor effect of sunitinib by inhibiting proliferation and inducing G0/G1/S phase arrest in malignant melanoma
Source: Oncotarget. 2017 Nov 25;9(1):802–11. doi: 10.18632/oncotarget.22696 (PMC5787512; doi:10.18632/oncotarget.22696)
Supplement: Supplementary file 1 [file oncotarget-09-802-s001.pdf]

Propranolol enhanced the anti-tumor effect of sunitinib by inhibiting proliferation and inducing G0/G1/S phase arrest in malignant melanoma

SUPPLEMENTARY MATERIALS

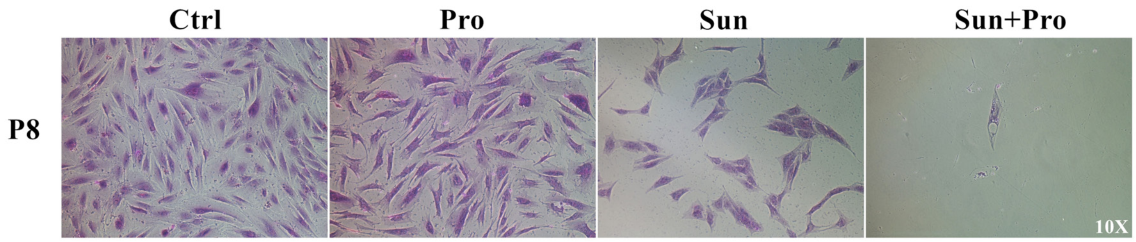

Supplementary Figure 1: C-PST significantly inhibited P8 cell line proliferation in the morphological changes.

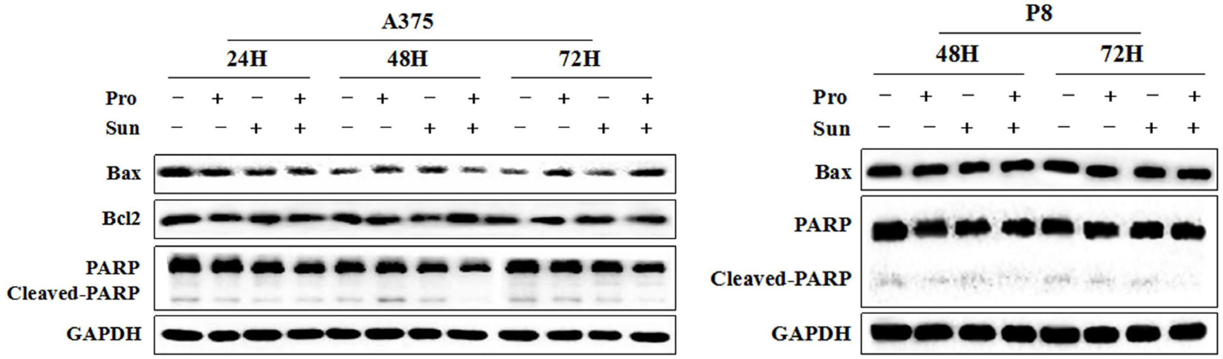

Supplementary Figure 2: Cell apoptosis related western blotting analysis of C-PST in malignant melanoma cells. C-PST did not induce the obviously change in the levels of Bax, Bcl2, Cleaved-PARP by treated with control group, Pro 50 µM, Sun 2.5 µM, C-PST and in (left) A375 cells and (right) P8 cells.

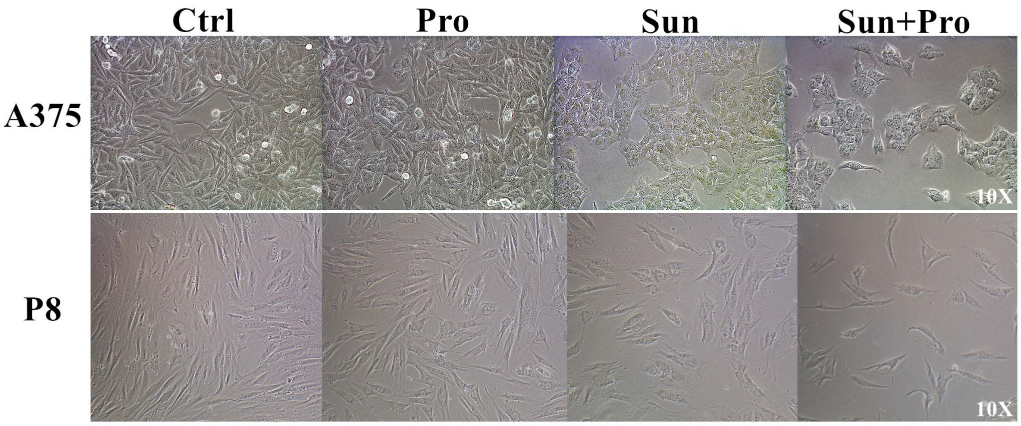

Supplementary Figure 3: The morphology changes after sunitinib, propranolol and combination treatment in melanoma cell lines for 48 hours. Sun: 2.5 µM Pro: 50 µM. Seed tumor cell at 50% confluent on Day 1. Upper panel: A375 cell Down panel: P8 cell.

Supplementary Table 1: Cell-cycle analysis of A375 and P8 cell lines

Cell distribution (%)		24H			48H		
		**G0/G1 phase**	**S phase**	**G2/M phase**	**G0/G1 phase**	**S phase**	**G2/M phase**
A375	Ctrl	63.0±4.2[*]	31.2±3.6[*]	6.7±0.2[*]	74.5±1.9[*]	19.5±1.3[*]	6.6±2.0[*]
	Pro	68.1±3.2[*]	24.3±3.8[*]	8.4±0.1[*]	73.4±1.2[*]	20.0±2.6[*]	6.4±1.1[*]
	Sun	73.0±2.6[*]	20.5±3.0	7.8±0.2[*]	79.2±1.7[*]	19.5±2.0[*]	1.6±0.2
	Sun+ Pro	82.7±2.1	14.1±1.2	3.7±0.3	92.4±0.9	6.1±0.9	1.5±0.3
	P value[#]	< 0.0001	0.0009	< 0.0001	< 0.0001	< 0.0001	0.0006
P8	Ctrl	53.3±4.0[*]	43.7±3.0[*]	3.2±1.1[*]	70.9±2.4[*]	19.8±1.3[*]	9.3±1.1
	Pro	65.6±2.0[*]	30.9±1.0[*]	3.5±1.2[*]	67.6±0.7[*]	17.8±2.7[*]	14.6±1.2[*]
	Sun	68.6±3.0[*]	29.1±8.2[*]	2.3±1.0	77.8±1.4[*]	7.5±0.9	14.9±2.0[*]
	Sun+ Pro	83.4±1.5	15.6±1.0	1.0±0.1	85.6+2.1	3.9±1.7	10.8±1.0
	P value[#]	< 0.0001	0.0004	0.0288	< 0.0001	< 0.0001	0.0018

[#] P value for overall comparison using one-way ANOVA.
[*] Significant difference in comparison with Sun + Pro, using Dunnett's multiple comparison test (P<0.05).
Data represent means (n=3)±SD.
